# Emergence of oxacillinase-181 carbapenemase-producing diarrheagenic *Escherichia coli* in Ghana

**DOI:** 10.1080/22221751.2021.1920342

**Published:** 2021-05-04

**Authors:** Isaac Prah, Alafate Ayibieke, Samiratu Mahazu, Chihiro Tani Sassa, Takaya Hayashi, Shoji Yamaoka, Toshihiko Suzuki, Shiroh Iwanaga, Anthony Ablordey, Ryoichi Saito

**Affiliations:** a Department of Molecular Microbiology, Tokyo Medical and Dental University (TMDU), Tokyo, Japan; b Department of Molecular Virology, Tokyo Medical and Dental University (TMDU), Tokyo, Japan; c Department of Clinical Laboratory, Tokyo Medical and Dental University Medical Hospital, Tokyo, Japan; d Department of Bacterial Pathogenesis, Tokyo Medical and Dental University (TMDU), Tokyo, Japan; e Department of Environmental Parasitology, Tokyo Medical and Dental University (TMDU), Tokyo, Japan; f Bacteriology Department, Noguchi Memorial Institute for Medical Research, University of Ghana, Accra, Ghana

**Keywords:** ST410, oxacillinase-181, diarrheagenic *E. coli*, IncX3, IncFIC(FII) and B4/H24RxC clade

## Abstract

The emergence and spread of carbapenemase-producing bacteria are serious threats to public health. We characterized two OXA-181-producing *Escherichia coli* isolates from pediatric patients with diarrhea from Ghana. *bla*_OXA_-_181_ was localized on the self-conjugative IncX3-containing plasmid in the *E. coli* ST410 isolate, belonging to an emerging lineage, and an IncFIC(FII)-containing plasmid in *E. coli* ST940. The *bla*_OXA-181_-*qnrS1* region was found on the IS*26* composite transposon, which contained a 366-bp deletion in the region encoding the Rep A protein for the IncX3-containing plasmid. The IncFIC(FII) plasmid was novel and integrated with an approximately 39-kb IncX1 plasmid through conjugal transfer. Both plasmids clustered close to plasmids from Switzerland. To the best of our knowledge, this is the first report describing the presence of an IncX3 plasmid containing *bla*_OXA-181_ in strains closely related to the B4/H24RxC clade in Africa, suggesting its emergence and the need to strengthen antimicrobial resistance surveillance.

## Introduction

^.^ Diarrheagenic *Escherichia coli* (DEC) are important causes of gastroenteritis in children from developing countries and are associated with high antibiotic resistance [1]. Various clinically important β-lactamases such as class A and B carbapenemases have been detected among DEC isolates [2]. Recently, a class D OXA-48-like carbapenemase was reported in Italy in a pediatric patient with diarrhea [3]. There are 14 variants of OXA-48-like enzymes (OXA-48, −162, −163, −181, −199, −204, −232, −244, −245,−247, −370, −405, −436, and −484) that are typically found in *Enterobacteriaceae* [4]. These variants exhibit geographical associations, and their proliferation is mediated by the spread of both successful clones and horizontal dissemination [4]. OXA-181 carbapenemase is the second most common OXA-48-like variant globally, and epidemiological evidence has linked this variant to the Indian subcontinent [4,5]. Dominant OXA-181-producing *E. coli* clones include ST410 and ST940 [6]. ST410 shows similarity to the ST131 pandemic lineage as a specific clade, B4/H24RxC, arose by acquisition of the IncX3 plasmid carrying *bla*_OXA-181_ [7,8], warranting its continuous monitoring to prevent spreading. Diverse plasmid types have been found to drive *bla_O_*_XA-181_ in different species [9–12] but is mostly driven by IncX3 plasmids, suggesting the potential of these plasmids as vehicles in the worldwide dissemination of OXA-181 [3,11,13,14].

A recent study by Labi and colleagues revealed a significant proportion of OXA-181-producing *Klebsiella spp*. among a neonatal population in a tertiary hospital in Ghana [15,16]. This finding suggests that the prevalence of *bla_O_*_XA-181_ is underreported in the general population, as most clinical laboratories are not equipped for its detection. Because this screening technique is not widely available in Ghana, data on *bla_O_*_XA-181_ is limited, particularly for other reservoirs of *bla_O_*_XA-181_ and its genetic environment.

We previously characterized DEC strains recovered from pediatric patients with diarrhea in the western region of Ghana [17]. This study was performed to evaluate the antibiotic resistance of these recovered *E. coli* isolates and use whole-genome sequencing to characterize two isolates harbouring *bla_O_*_XA-181_.

## Materials and methods

### Ethnical statements

This study was approved by the ethics committee of Noguchi Memorial Institute for Medical Research, University of Ghana (FWA 00001824) and Faculty of Medicine, Tokyo Medical and Dental University (M2017-208). Informed parental consent was obtained from the parent or guardian of each participant.

### Bacterial strains

We evaluated 62 *E. coli* isolates that had been pre-characterized in a previous study as DEC (*n* = 37) and non-DEC (*n* = 25) strains (Supplementary dataset 1) [17]. These strains were recovered from stools samples of pediatric patients with diarrhea from the western region of Ghana.

### Antimicrobial susceptibility testing and β-lactamase-encoding gene screening

The antimicrobial susceptibility of these 62 *E. coli* isolates to a panel of 16 antimicrobials (piperacillin, cefazolin, cefotaxime, ceftazidime, cefepime, cefpodoxime, sulbactam/ampicillin, aztreonam, gentamicin, amikacin, minocycline, fosfomycin, levofloxacin, sulfamethoxazole/trimethoprim, imipenem, and meropenem) on a DP31 dry plate (Eiken Chemical Co., Tokyo, Japan) was evaluated by broth microdilution following the manufacturer’s instructions. The minimum inhibitory concentration (MIC) values were interpreted according to the guidelines described in the Clinical Laboratory and Standards Institute (30th Edition). Quality control using *E. coli* ATCC 25922 was also performed.

Genomic DNA for strains resistant to any β-lactam drugs screened was extracted using a Nucleo Spin Tissue kit (Takara Bio, Shiga, Japan) as described previously [17]. The presence of *bla*_TEM_, *bla*_SHV_, *bla*_OXA-1_-like, and *bla*_CTX-M_ group 1 were screened by PCR as previously described [18]. *bla*_OXA-48_-like genes and other carbapenemase genes such as *bla*_VIM_, *bla*_IMP_, *bla*_KPC_, and *bla*_NDM_ were also analyzed [18,19]. In-house strains were used as positive controls.

### Conjugation experiments

Conjugal transfer of a *bla*_OXA48_-like gene detected was assessed by the agar mating method as described previously with some modifications [19,20]. A 1:1 donor to recipient (rifampicin-resistant *E*. *coli* strain, C600) ratio of 1 mL of mixed cell cultures was prepared. The mixtures were centrifuged at 10,000 ×*g* for 3 min, and 800 μL of the supernatant was discarded. The remaining 200 μL of each mix was vortexed and transferred onto tryptic soy agar plates. The plates were incubated 37°C for 18–20 h, after which the cells were collected into 1 mL of 0.9% sterile physiological saline. Ten-fold dilutions were plated on Bromo Thymol Blue lactose agar plates (Sigma Aldrich, St. Louis, MO, USA) supplemented with 50 μg/mL of rifampicin to select recipients and 8 μg/mL of ampicillin, 4 μg/mL of sulbactam, and 50 μg/mL of rifampicin to select transconjugants. Transfer frequencies were expressed as the number of transconjugant colonies (TC) per recipient colonies (rec) formed after mating.

### S1-nuclease pulsed-field gel electrophoresis and Southern blot analysis

To determine the localization of *bla*_OXA48_-like genes in the genome, S1-nuclease pulsed-field gel electrophoresis and Southern blotting were performed as described previously [19]. Briefly, genomic DNA from donor cells as well as recipient and transconjugants strains were prepared in agarose plugs and digested with S1 nuclease (Takara Bio). Partially digested DNA molecules were separated using the CHEF-mapper XA system (Bio-Rad, Hercules, CA, USA). After electrophoresis, DNA in the pulsed-field gel (after a section was stained with EDTA) were transferred onto a Hybond N^+^ membrane (GE Healthcare, Little Chalfont, UK) and hybridized with a digoxigenin-labelled *bla*_OXA-48_-like gene probe generated from a published primer set [18]. The probe signal was detected by digoxigenin high-prime DNA labelling with a detection starter kit (Roche Diagnostics, Basel, Switzerland) according to the manufacturer’s instructions.

### Whole-genome sequencing and comparative analysis of *bla*_OXA-48_-like-producing *E. coli* isolates

Genomic DNA from the *bla*_OXA-48_-like-producing *E. coli* isolates (1EC187 and 1EC213) was prepared using a NucleoBond HMW DNA Kit (Macherey-Nagel, Düren, Germany) following the manufacturer's instructions. Sequencing libraries for Illumina short reads were generated with a Nextera DNA Library Preparation Kit, and sequencing was conducted on an Illumina Miseq (San Diego, CA, USA). Low-quality reads (quality below Q <30 and length <10 bp) were filtered out. A Nanopore MinION was used for long-read sequencing (Oxford Nanopore Technologies, Oxford, UK). Libraries were constructed using an SQK LSK108 ligation sequence kit and sequenced with a FLO MIN106 R9.41 Flow Cell. Reads with low qualities (quality below Q <10 and length <500 bp) were filtered out. The yields of Illumina Miseq and Nanopore MinION sequencing data are shown in Supplementary Table S1. *De novo* hybrid assembly of both Illumina short reads and Nanopore MinION long reads was performed using Unicycler v0.4.8 [21]; Table S2 shows the *de novo* assembly statistics. Sequence annotation was performed using RAST and BLASTP/BLASTN [22,23]. The Center for Genomic Epidemiology online tools (https://cge.cbs.dtu.dk) such as ResFinder, PlasmidFinder, MLST, pMLST, Fimtyper, SerotypeFinder and ISfinder (https://www-is.biotoul.fr/) were utilized to detect resistance genes, plasmid replicon type, multilocus sequence type (ST), plasmid type, FimH type, serotype, and mobile elements, respectively. The genetic structures of the *bla*_OXA-181_-containing plasmids (pEC187_2-OXA-181 and pEC213_1-OXA-181) were compared and visualized using EasyFig v2.1 [24]. Phylogenetic analysis of these two plasmids and 11 other *bla*_OXA-181_ circulating plasmids available from GenBank (showing the highest homology to these plasmids) was conducted using MEGA X software [25].

To determine whether the ST410-producing isolate 1EC187 belongs to the B4/H24RxC clade, ST410 genome sequences with short reads available from GenBank (*n* =** **73; Supplementary dataset 2, accessed on November 24, 2020) were retrieved from the NCBI Sequence Read Archive database. These sequences were selected based on published studies on ST410 phylogeny [7,8]. The chromosomal sequence of ST410 strain belonging to B4/H24RxC, SCEC020026 (accession no. CP034958), available from GenBank was used as a reference for mapping. The Sequence Read Archive of 73 *E. coli* ST410 strains and chromosomal sequence of 1EC187 (accession no. CP061108) were aligned to the reference using Snippy v4.3.6 [26] with default settings. The aligned pseudo-genomes were cleaned using Snippy integrated scripts. To obtain a precise phylogeny, single-nucleotide polymorphism (SNP) sites residing in recombination regions were masked, and the recombination-corrected phylogenetic tree was inferred using Gubbins v 2.3.4 [27]. The Iq tree was used to construct a phylogenetic tree from the resulting file using 1000 bootstrapping replicates [28]. The ST410 phylogenetic tree was visualized and annotated using iTOL v4 [29].

### Whole-genome sequencing of transconjugant strain Tc1EC213

Genomic DNA of Tc1EC213 was extracted using a Nucleo Spin Tissue kit and sequenced by Nanopore MinION long read technology. A sequence library was constructed with the SQK LSK109 ligation sequence kit and run on a FLO MIN106 D Flow Cell. Reads with low qualities were filtered out and *de novo* assembly was conducted using Unicycler v0.4.8 [21].

## Results

### Antimicrobial susceptibility and β-lactamase-encoding gene screening

Overall, both DEC and non-DEC isolates were susceptible (100%) to carbapenems and fosfomycin but showed varying degrees of susceptibility to the other drugs. They expressed high resistance to piperacillin (73%, DEC; 72%, non-DEC) and sulfamethoxazole/trimethoprim (86.5%, DEC; 72%, non-DEC) (Table 1). The percentage resistance of DEC isolates to the third-generation cephalosporins, tetracyclines, and fluoroquinolones were 24.3% (cefotaxime), 27% (minocycline), and 16.2% (levofloxacin), respectively (Table 1).

**Table 1.** Antibiotic resistance profile of diarrheagenic *E. coli* (DEC) and non-diarrheagenic *E. coli* (non-DEC) isolates from the western region of Ghana.

Antibiotics	All isolates (*n* = 62)				DEC^1^ isolates (*n* = 37)				Non-DEC isolates (*n* = 25)			
	MIC (μg/mL)			% R	MIC (μg/mL)			%R	MIC (μg/mL)			%R
	Range	MIC_50_	MIC_90_		Range	MIC_50_	MIC_90_		Range	MIC_50_	MIC_90_	
Piperacillin	1–>64	>64	>64	72.6	1–>64	>64	>64	73	1–>64	>64	>64	72
Cefazolin	1–>16	2	>16	30.6	1–>16	2	>16	35.1	1–>16	4	>16	36
Cefotaxime	≤0.5–>32	≤0.5	>32	19.3	≤0.5–>32	≤0.5	>32	24.3	≤0.5–>32	≤0.5	32	12
Ceftazidime	≤0.5–>16	≤0.5	16	16.1	≤0.5–>16	≤0.5	16	18.9	≤0.5–>16	≤0.5	8	8
Cefepime	≤0.5–>16	≤0.5	>16	14.5	≤0.5–>16	≤0.5	>16	21.6	≤0.5–>16	≤0.5	8	4
Cefpodoxime	≤1–>4	≤1	>4	19.3	≤1–>4	≤1	>4	24.3	≤1–>4	≤1	>4	12
Sulbactam/ampicillin	≤2/4–>8/16	4/8	>8/16	14.5	≤2/4–>8/16	4/8	>8/16	16.2	≤2/4–>8/16	8/16	>8/16	12
Aztreonam	≤0.5–>16	≤0.5	>16	19.3	≤0.5–>16	≤0.5	>16	24.3	≤0.5–>16	≤0.5	16	12
Gentamicin	≤0.25–>8	0.5	2	0	≤0.25–4	1	2	0	≤0.25–>8	0.5	1	0
Amikacin	≤1–32	2	8	0	≤1–32	4	8	0	≤1–32	2	8	0
Minocycline	0.5–>8	4	>8	27.4	0.5–>8	4	>8	27	2–>8	4	>8	28
Fosfomycin	≤32	≤32	≤32	0	≤32	≤32	≤32	0	≤32	≤32	≤32	0
Levofloxacin	≤0.25–>4	≤0.25	>4	12.9	≤0.25–>4	≤0.25	>4	16.2	≤0.25–4	≤0.25	1	8
Sulfamethoxazole/ trimethoprim	≤9.5/0.5–>38/2	>38/2	>38/2	80.6	≤9.5/0.5–>38/2	>38/2	>38/2	86.5	≤9.5/0.5–>38/2	>38/2	>38/2	72
Imipenem	≤0.25–0.5	≤0.25	≤0.25	0	≤0.25	≤0.25	≤0.25	0	≤0.25–0.5	≤0.25	≤0.25	0
Meropenem	≤0.25	≤0.25	≤0.25	0	≤0.25	≤0.25	≤0.25	0	≤0.25	≤0.25	≤0.25	0

**Abbreviation**: % R, percentage resistance

Apart from the *bla*_OXA-48_-like gene, no other carbapenemase genes were detected. An ETEC isolate (1EC187) and non-DEC isolate (1EC 213) were positive for the *bla*_OXA-48_-like gene. The details of other β-lactamase genes are shown in Supplementary dataset 1.

### Plasmid-mediated transfer of *bla*_OXA-48_-like

Plasmids harbouring *bla*_OXA 48_-like were successfully transferred from donor cells (1EC187 and 1EC213) to recipient cell, *E. coli* C600, via conjugal transfer. The observed transfer frequencies were 2.4 × 10^−3^ and 1.4 × 10^−4^ TC/rec respectively. *bla*_OXA-48_-like was localized on a ∼50-kbp plasmid in both 1EC187 and its transconjugant, Tc1EC187, but with its transconjugant, 1EC213, Tc1EC213 showed a different plasmid size (Figure S1). To gain insight into the size difference between the donor and this transconjugant plasmid, we sequenced Tc1EC213 using Nanopore. From the assembled data, the size of the *bla*_OXA-48_-like containing plasmid (pTc1EC213_1-OXA-181) was 135,193 bp (Table S2). pTc1EC213_1-OXA-181 showed an increased size because it had acquired an extra plasmid from 1EC213. Compared to their recipient cell, the transconjugants (Tc1EC187 and Tc1EC213 cells) were susceptible to most of the drugs tested (Table S3). Tc1EC187 was resistant to piperacillin and cefazolin but showed intermediate resistance to sulbactam/ampicillin. Tc1EC213 was also resistant to piperacillin and sulbactam/ampicillin but showed intermediate resistance to cefazolin (Table S3).

### Whole-genome characterization of 1EC187 and 1EC213

**Figure 1.**
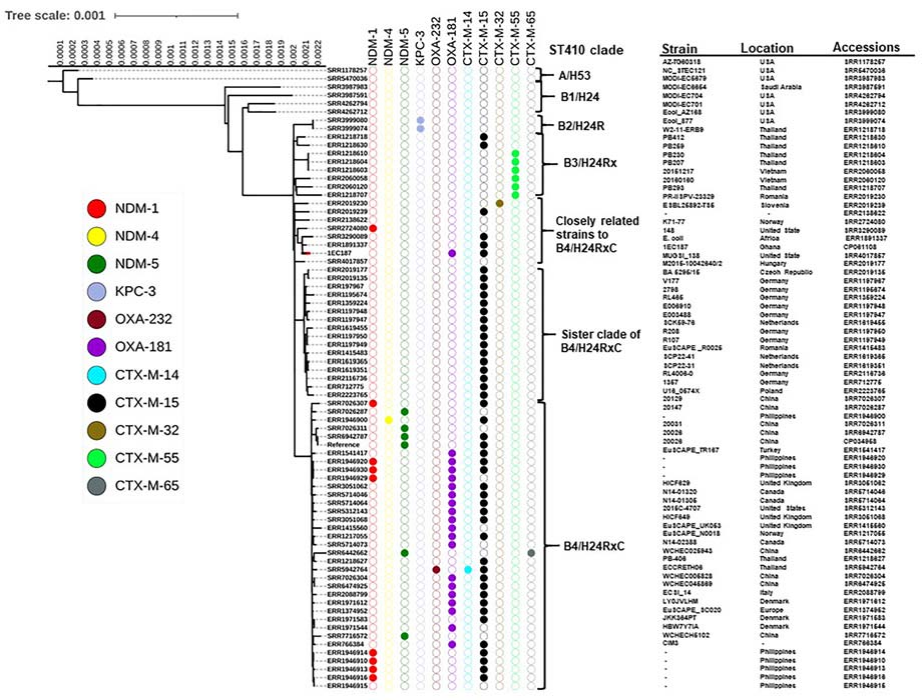
Rooted maximum likelihood phylogenetic tree of 75 *Escherichia coli* sequence type 410 genomes. A reference-based SNP phylogeny followed by filtering of recombination sites was constructed. The phylogeny of 1EC187 to the other ST410 genomes is indicated by a red branch colour. The presence of carbapenemase (*bla*_NDM-1_, *bla*_NDM-4_, *bla*_NDM-5_, *bla*_KPC-3_, *bla*_OXA-181_, *bla*_OXA-232_) and ESBL (*bla*_CTX-M-14_, *bla*_CTX-M-15_, *bla*_CTX-M-32_, *bla*_CTX-M-55_, *bla*_CTX-M-65_) genes in these genomes are represented by a shaded circle. The strain names, location, and accession numbers of these ST410 strains are also shown.

To provide insight into other *bla*_OXA-181_ reservoirs circulating in Ghana, the two *bla*_OXA-48_-like containing isolates were evaluated by whole-genome sequencing. The genomic size of 1EC187 and 1EC213 were 5,054,351 and 4,991,513 bp, respectively. The genome of 1EC187 consisted of 4,847,148 bp chromosomal DNA and four circular plasmids with a GC content of 50.5% (Table S4), whereas 1EC213 had 4,746,359 bp chromosomal DNA and six circular plasmids with a GC content of 50.6% (Table S4). 1EC187 belonged to ST410 with serotype O8:H9 and fimH24 type, whereas 1EC 213 belonged to ST940 and serotype Ont:H4. To determine whether the ETEC ST410 strain belongs to the B4/H24RxC clade, a reference-based SNP phylogeny of 1EC187 and other ST410 strains was constructed. In phylogenetic analysis, 1EC187 did not cluster with B4/H24RxC clade strains but rather with a strain (accession no. ERR1891337) closely related to the B4/H24RxC clade (Figure 1).

Both isolates expressed a variant of ESBL genotypes (1EC187, *bla*_CTX-M-15_) and (1EC213, *bla*_TEM-35_) (Table 2). Most resistance genes in these genomes were plasmid-mediated, and *bla*_OXA-181_ were borne by plasmids of different incompatibility groups (Table 2).

**Table 2:** Variability in antibiotic resistance genes in the genome of OXA-181-producing isolates

			Isolate ID				
			1EC187			1EC213	
			Chromosome	pEC187_1	pEC187_2-OXA-181	Chromosome	pEC213_1-OXA-181
Sequence type			ST410			ST940	
Plasmid incompatibility group			-	IncFIA	IncX3	-	IncFIC(FII)
pMLST				F-:A1:B58	-		F-:A-:B12
Antibiotic resistance gene							
	Quinolone	*aac(6')-lb-cr*	+				
		*qnrS1*			+		+
	Sulfonamide	*sul2*		+			+
	Trimethoprim	*dfrA14*		+		+	
	Tetracycline	*tet(B)*		+		+	
	β-lactam	*bla*_CTX-M-15_	+				
		*bla*_OXA-1_	+			+	
		*bla*_OXA-181_			+		+
		*bla*_TEM-1B_		+			
		*bla*_TEM-35_					+
	Macrolide	*mdf(A)*	+			+	
		*mph(A)*		+			
	Phenicol	*catA1*		+		+	
	Aminoglycoside	*aac(6’)-lb-cr*	+				
		*aph(3’’)-lb*		+			+
		*aph(6)-ld*		+			+

### Genetic and phylogenetic analyses of the two *bla*_OXA-181_-containing plasmids

**Figure 2.**
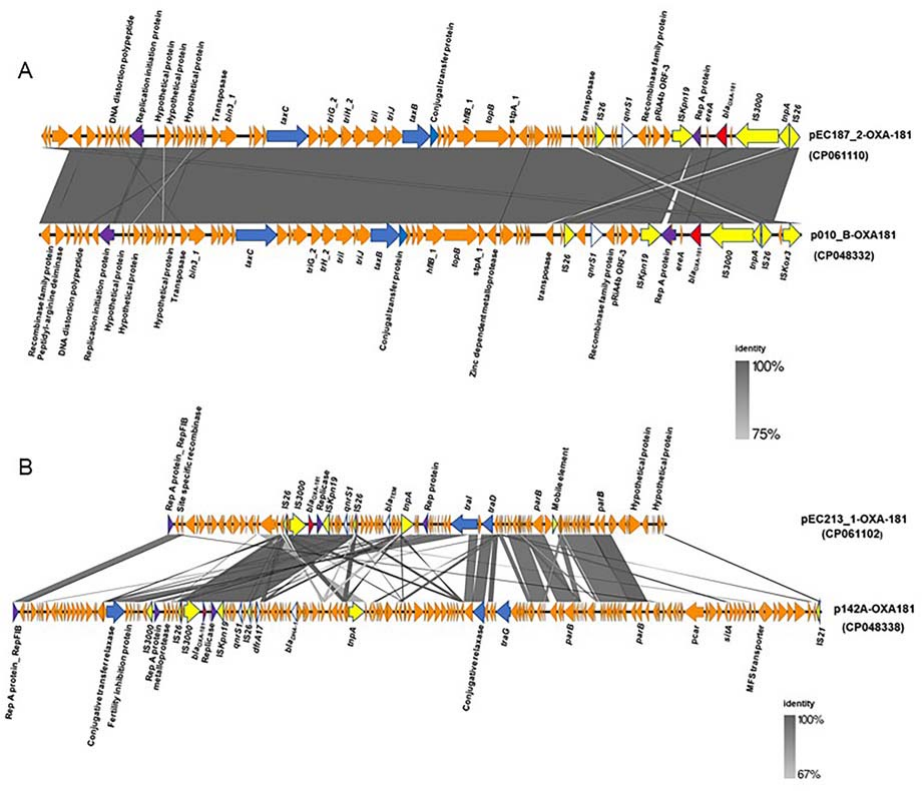
Linearized backbone structure comparison of OXA-181-containing plasmids. (A). IncX3 type plasmids containing OXA-181, pEC187_2-OXA-181, and p010-B_OXA181 (CP048332). (B) IncFIC(FII) type plasmid, pEC213_1-OXA-181, and IncFII type plasmid, p142A-OXA181. Similar features are represented by the same colour. Replicon, conjugal transfer genes, mobile elements, antibiotics resistance genes, *bla*_OXA-181_ and other genes are represented by violet, blue, yellow, white, red, and orange colours, respectively. Direction of the coding sequences are indicated by the direction of the arrow. The grey colour represents the region of similarity between the plasmids.

**Figure 3.**
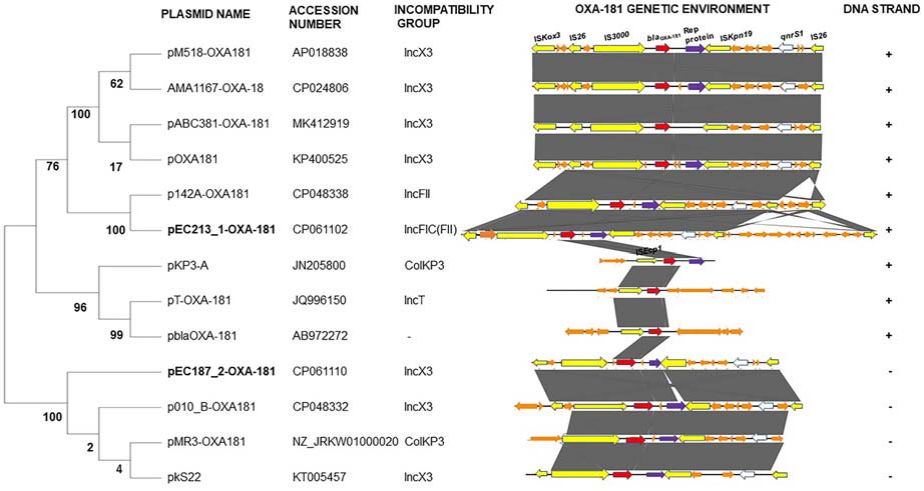
Evolutionary analysis of pEC187_2-OXA-181, pEC213_1-OXA-181, and 11 OXA-181-containing plasmids (having highest homology to these two plasmids) performed by the maximum likelihood method. A phylogenetic tree was constructed using 1,000 bootstrap replicates, and bootstrap values are shown next to the branches. The plasmid names and their accession numbers are provided along with the plasmid incompatibility group. A schematic diagram of the *bla*_OXA-181_ genetic environment (*bla*_OXA-181_ surrounding region ranging from 7 to 23.5 kbp) is also shown. Similar features are represented by the same colour. Replicon, mobile elements, antibiotics resistance genes, *bla*_OXA-181_, and other genes are represented by violet, yellow, white, red, and orange colours, respectively. The grey colour represents the region of similarity between the plasmids. DNA strand direction is shown at the extreme right. Positive and reverse strands are labelled with the symbols “+” and “−”, respectively.

**Figure 4.**
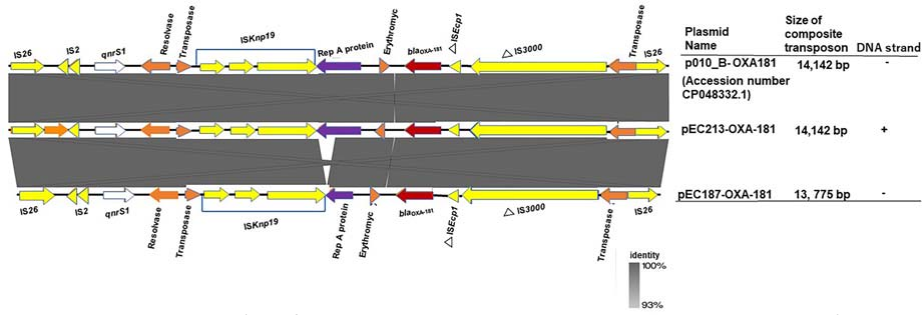
Linearized comparison of IS26 flanked composite transposons carrying *bla*_OXA-181_ and *qnrS1*. Similar features are represented by the same colour. Replicon, mobile elements, antibiotics resistance genes, *bla*_OXA-181_, and other genes are represented by violet, yellow, white, red, and orange colours, respectively. The triangle symbolizes truncated features, whereas the grey colour represents the region of similarity between the plasmids. DNA strand direction is shown at the extreme right. Positive and reverse strands are labelled with the symbols “+” and “-”, respectively.

A nucleotide blast search against the nr/nt database showed that the 51,113-bp IncX3 plasmid containing OXA-181 (pEC187_2-OXA-181, accession no. CP061110) shared 100% query coverage and identity with 17 plasmids including p010_B-OXA181 (accession no. CP048332). pEC187_2-OXA-181 shared a similar backbone structure with p010_B-OXA181 (Figure 2), and the orientation of *bla*_OXA-181_-*qnrS1* was identical to those of p010_B-OXA181, pMR3-OXA181, and pKS22 (Figure 3). The genetic environment of *bla*_OXA-181_ and the quinolone resistance gene *qnrS1* contained a similar IS*26*-flanked composite transposon also shared by p010_B-OXA181 but with a 366-bp deletion in Rep A protein (Figure 4). In contrast, the 95,832-bp IncFIC(FII) plasmid containing OXA-181 (pEC213_1-OXA-181, accession no. CP061102) showed the highest homology with only 49% query coverage and 99.96% identity to p142_A-OXA181 (accession no. CP048338). These two plasmids shared high background similarity around the OXA-181-containing region (Figure 2). The same IS*26*-flanked composite transposon found in p010_B-OXA181 was detected in pEC213_1-OXA-181 (Figure 3). pEC213_1-OXA-181 also carried various drug resistance genes such as *sul2*, *bla*_TEM*-*35_, *aph(3’’)-lb*, and *aph(6)-ld* (Table 2).

pEC187_2-OXA-181 and pEC213_1-OXA1-81 clustered differently from each other in phylogenetic analysis (Figure 3). pEC187_2-OXA-181 showed a closer phylogenetic relationship with plasmids from Switzerland (CP048332, KT005457) and the United States (NZ_JRKW01000020), whereas pEC213_1-OXA-181 also clustered with a plasmid from Switzerland (CPO48338).

## Discussion

Administration of antibiotics to treat pediatric acute diarrhea may lead to the promotion of antimicrobial resistance, particularly when used to treat non-bacterial illnesses [30]. We determined the antibiotic susceptibility profile of 62 *E. coli* isolates from pediatric patients with diarrhea in the western region of Ghana.

We observed a high resistance rate against sulfamethoxazole/trimethoprim among DEC (86.5%) and non-DEC isolates (72%). A similar resistance rate was reported in Peru and Iran among DEC isolates [1,31]. Sulfamethoxazole/trimethoprim is typically used in diarrhea empiric therapy while awaiting microbiological investigation [32]; this selective pressure may account for the high resistance to sulfamethoxazole/trimethoprim. The common resistance pattern observed was piperacillin/sulfamethoxazole/trimethoprim/minocycline (penicillin/co-trimoxazole/tetracycline), which was similar to previous findings [31].

The OXA-181-encoding gene was first reported in 2007 and was subsequently identified in several countries, including its recent detection in Ghana [15,16,33]. It is mainly found in *E. coli* and *K. pneumoniae* strains with an increasing prevalence [33]. The IncX3 plasmid pEC187_2-OXA-181 harbouring *bla*_OXA-181_ showed the same nucleotide identity as 17 other plasmids from 9 different countries (Czech Republic, Denmark, Myanmar, Lebanon, Japan, United Arab Emirates, United Kingdom, Switzerland and United States). This supports its conserved nature and important role in the dissemination of *bla*_OXA-181_. *bla*_OXA-181_ on pEC187_2-OXA-181 exhibited a similar genetic context as previously described [9,34,35]. It was flanked upstream by IS*Ecp1* and IS*3000* (both truncated) and downstream by the replication initiation protein (Rep A), IS*Knp19*, and *qnrS1*, which occurred within the IS*26* composite transposon. This composite transposon differed from that previously described by a 366-bp deletion in Rep A protein.

pEC187_2-OXA-181 was found in the *E. coli* ST410 lineage, which is predominantly associated with *bla*_OXA-181_ [6]. Apart from pEC187_2-OXA-181, 1EC187 contained the IncFIA plasmid harbouring several antibiotics genes and chromosomal-mediated *bla*_CTX-M-15_ (Table 2). MDR *E. coli* belonging to the ST410 lineage are emerging as high-risk clones globally, particularly the B4/H24RxC clone [7,8,36]. This specific clone arose from the background population of ST410 by acquiring an IncX3 plasmid carrying *bla*_OXA-181_. Reference-based SNP phylogeny analysis of 1EC187 revealed a close genetic distance to a strain that is also of African origin (Figure 1) and that these strains form part of a cluster that has been characterized as emerging [8]. Feng and colleagues [8] showed that these emerging ST410 strains lack IncX3-containing *bla*_OXA-181_. Our results suggested the likely spread of the IncX3-containing *bla*_OXA-181_ gene in these strains.

The other *bla*_OXA-181_ detected in this study was on a self-conjugative IncFIC (FII) plasmid (pEC213_1-OXA-181). The *bla*_OXA-181_–*qnrS1* region was also flanked by the IS*26* composite transposon (Figure 4). pEC213_1-OXA-181 shared low resemblance with other OXA-181 circulating plasmids including pEC187_2-OXA-181. Phylogenetic analysis involving these two plasmids exhibited no evolutionary relatedness (Figure 3), suggesting important roles for these different plasmids in spreading *bla*_OXA-181_ in the region. pEC213_1-OXA-181 harboured several resistance genes, and this novel plasmid may exacerbate the spread of antibiotic resistance genes.

**Figure 5.**
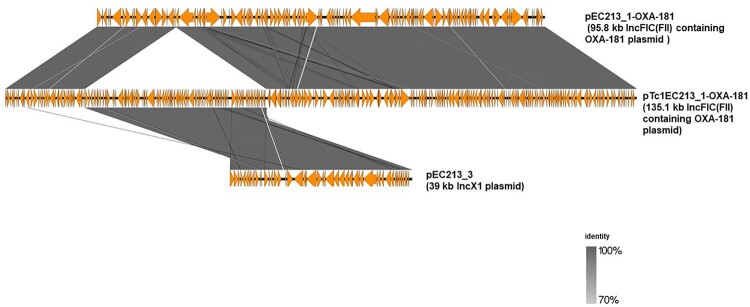
Linearized comparison of pEC213_1-OXA-181 and it co-integrate transconjugant form (pTc1EC213_1-OXA-181). An approximately 39-kb IncX1 plasmid (pEC213_3) was integrated on OXA-181-containing IncFIC (FII) plasmid (pEC213_OXA-181) in a conjugal transfer.

In conjugal transfer of 1EC213 and *E. coli* C600 followed by sulbactam/ampicillin and rifampicin selection, the resulting transconjugant plasmid form (pTc1EC213_1-OXA-181) was a co-integrate of pEC213_1-OXA-181 and pEC213_3, an IncX1-containing plasmid also from 1EC213 (Figure 5). Fusion of these plasmids may have been mediated by a *dif*-like sequence on both plasmids. This facilitated the inter molecular site-specific recombination reaction resulting in an increased plasmid size, as shown in Figure S1 [37]. Similarly, pEC213_1-OXA-181 was in *E. coli* lineage (ST940), which is associated with *bla*_OXA-181_ [6].

In summary, our study provides insight into the *E. coli* lineage containing OXA-181 in Ghana. To the best our knowledge, this is the first study to describe conjugative OXA-181-containing plasmids from these strains. The detection of emerging and closely related strains to the B4/H24RxC clone harbouring the IncX3 plasmid from Africa demonstrate the necessity of strengthening antimicrobial resistance surveillance.

## Supplementary Material

Supplemental Material

Supplementary_dataset_2_revised_final.xlsx

Supplementary_dataset_1_revised_final.xlsx

Figure_RC3.jpg

Figure_RC2.jpg

Figure_RC.1.jpg
