# Supplementary material for: Emergence of oxacillinase-181 carbapenemase-producing diarrheagenic Escherichia coli in Ghana
Source: Emerg Microbes Infect. 2021 May 4;10(1):865–73. doi: 10.1080/22221751.2021.1920342 (PMC8110189; doi:10.1080/22221751.2021.1920342)
Supplement: Supplemental Material [file TEMI_A_1920342_SM5720.pdf]

Supplementary Files

Emergence of oxacillinase-181 carbapenemase-producing diarrheagenic *Escherichia coli* in Ghana

Isaac Prah[1,2], Alafate Ayibieke[1], Samiratu Mahazu[1,5], Chihiro Tani Sassa[3], Takaya Hayashi[2], Shoji Yamaoka[2], Toshihiko Suzuki[4], Shiroh Iwanaga[5], Anthony Ablordey[6], Ryoichi Saito[1]*

[1]Department of Molecular Microbiology, Tokyo Medical and Dental University (TMDU), Tokyo, Japan.

[2]Department of Molecular Virology, Tokyo Medical and Dental University (TMDU), Tokyo, Japan.

[3]Department of Clinical Laboratory, Tokyo Medical and Dental University Medical Hospital, Tokyo, Japan.

[4]Department of Bacterial Pathogenesis, Tokyo Medical and Dental University (TMDU), Tokyo, Japan.

[5]Department of Environmental Parasitology, Tokyo Medical and Dental University (TMDU), Tokyo, Japan.

[6]Bacteriology Department, Noguchi Memorial Institute for Medical Research, University of Ghana, Accra, Ghana.

*Corresponding author

Ryoichi Saito

Tel/Fax: +81-3-5803-5368

E-mail: r-saito.mi@tmd.ac.jp

Table S1: Filtered quality read statistics

Sample	Reads	Number of sequences	Sum of Length	Minimum Length	Average length	Maximum Length
1EC187	Miseq_1	1,779,417	264,553,378	31	148.7	152
	Miseq_2	1,779,417	264,467,085	25	148.6	152
	MinION	236,467	1,106,391,598	500	4,678.8	239,479
1EC213	Miseq_1	2,387,846	357,385,252	21	149.7	152
	Miseq_2	2,387,846	357,210,522	30	149.6	152
	MinION	224,413	572,027,648	500	2,549.0	190,672
TcEc213	MinION	129,434	620,717,367	1000	4795.6	40425

Table S2: *De novo* hybrid assembly statistics

Sample	Chromosome/ Plasmids	Length(bp)	Circular/ linear	GC (%)	Average read depth	Assembler
1EC187	chromosome	4,847,148	Circular	50.6	104.9	unicycler
	pEC187_1	112,598	Circular	52.9	40.2	unicycler
	pEC187_2-OXA-181	51,113	Circular	46.2	66	unicycler
	pEC187_3	41,391	Circular	45.4	77.7	unicycler
	Pec187_4	2,101	Circular	47.5	4227.6	unicycler
1EC213	chromosome	4,746,359	Circular	50.8	137.8	unicycler
	pEC213_1-OXA-181	95,832	Circular	51.6	68.6	unicycler
	pEC213_2	92,656	Circular	47.4	129.2	unicycler
	pEC213_3	39,019	Circular	40.4	196.8	unicycler
	pEC213_4	10,899	Circular	39.2	1382.8	unicycler
	pEC213_5	3,401	Circular	46.0	3110.7	unicycler
	pEC213_6	3,347	Circular	48.2	2604.1	unicycler

Table S3: Antibiotic susceptibility profile of donor, transconjugant, and recipient cells

Antibiotics	Minimum Inhibitory Concentration (MIC) µg/mL				
	1EC187	Tc1EC187	1EC213	Tc1EC213	C600
Piperacillin	>64	>64	>64	>64	4
Cefazolin	>16	8	16	4	2
Cefotaxime	>32	≤ 0.5	≤ 0.5	≤ 0.5	≤ 0.5
Ceftazidime	16	≤ 0.5	≤ 0.5	≤ 0.5	≤ 0.5
Cefepime	>16	≤ 0.5	≤ 0.5	≤ 0.5	≤ 0.5
Cefpodoxime	>4	≤ 1	≤ 1	≤ 1	≤ 1
Sulbactam/Ampicillin	>8/16	8/16	>8/16	>8/16	≤ 2/4
Aztreonam	>16	≤ 0.5	≤ 0.5	≤ 0.5	≤ 0.5
Gentamicin	4	<0.5	1	≤ 0.25	≤ 0.25
Amikacin	4	≤ 1	2	≤ 1	≤ 1
Minocycline	>8	1	8	1	1
Fosfomycin	≤ 32	≤ 32	≤ 32	≤ 32	≤ 32
Levofloxacin	>4	1	1	1	≤0.25
Sulfamethoxazole /trimethoprim	>38/2	≤ 9.5/0.5	>32/2	≤ 9.5/0.5	≤ 9.5/0.5
Imipenem	≤ 0.25	≤ 0.25	0.5	0.5	≤ 0.25
Meropenem	≤ 0.25	≤ 0.25	≤ 0.25	≤ 0.25	≤ 0.25

Table S4: Annotation statistics for 1EC187 and 1EC213

Name	Statistical values	
	1EC187	1EC213
Total Sequence length (bp):	5054351	4991513
Number of Sequences:	5	7
Longest Sequences (bp):	4847148	4746359
N50 (bp):	4847148	4746359
Gap Ratio (%):	0	0
GC content (%):	50.5	50.6
Number of CDSs:	4729	4739
Average Protein Length:	310.6	305.7
Coding Ratio (%):	87.2	87.1
Number of rRNAs:	22	22
Number of tRNAs:	90	95
Number of CRISPRs:	2	2

Figure S1

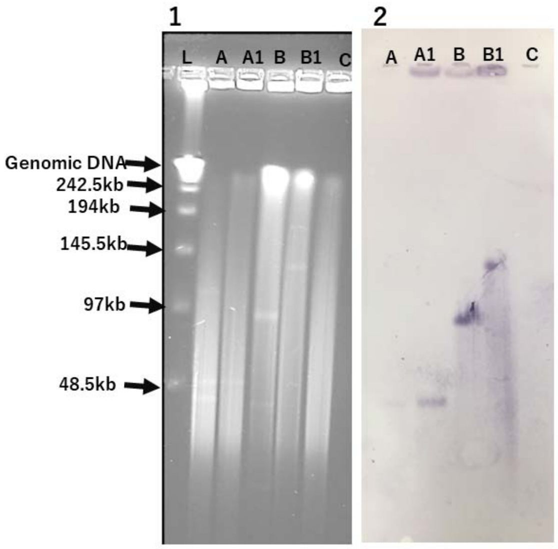

Figure S1: Localization of *bla*$_{OXA-48}$-like genes on mobile genetic elements. (**1**) S1 nuclease pulsed field gel electrophoretic profile of donors and their transconjugants harboring *bla*$_{OXA-48}$-like genes. Lane L is the lambda ladder; Lanes A & A1 (1EC 187, Tc1EC187); Lanes B & B1 (1EC 213, Tc1EC213); Lane C (C600, recipient cell) **(2**) Southern blot hybridization with *bla*$_{OXA\ 48}$-like gene probe. Lanes A–B1 showing signal bands corresponding to OXA 48-like-containing mobile elements.

Supplementary dataset 1

Strain ID	Year of Isolation	*E. coli* pathotype	Antibiotics (MIC; µg/mL)																Type of β- lactamase gene detected
			Piperacillin	Cefazolin	Cefotaxime	Ceftazidime	Cefepime	Cefpodoxime	Sulbactam/ Ampicillin	Aztreonam	Imipenem	Meropenem	Gentamicin	Amikacin	Minocycline	Fosfomycin	Sulfamethoxazole/ trimethoprim	Levofloxacin	
1EC47	2016	ETEC	4	2	≤0.5	≤0.5	≤0.5	≤1	≤2/4	≤0.5	≤0.25	≤0.25	0.5	2	4	≤32	≤9.5/0.5	≤0.25	None
1EC49	2016	ETEC	>64	2	≤0.5	≤0.5	≤0.5	≤1	4/8.	≤0.5	≤0.25	≤0.25	2	4	4	≤32	>38/2	≤0.25	*bla* $_{TEM}$
1EC70	2016	EAEC	>64	8	≤0.5	≤0.5	≤0.5	≤1	>8/16	≤0.5	≤0.25	≤0.25	≤0.25	2	1	≤32	>38/2	≤0.25	*bla* $_{TEM}$
1EC73	2017	ETEC	>64	>16	>32	>16	>16	>4	>8/16	>16	≤0.25	≤0.25	2	16	>8	≤32	>38/2	>4	*bla* $_{OXA-1}$-like , *bla* $_{CTX-M}$ group 1
1EC78	2017	EHEC	>64	>16	>32	>16	16	>4	>8/16	>16	≤0.25	≤0.25	2	32	>8	≤32	>38/2	>4	*bla* $_{OXA-1}$-like , *bla* $_{CTX-M}$ group 1
1EC84	2017	ETEC	>64	2	≤0.5	≤0.5	≤0.5	≤1	4/8.	≤0.5	≤0.25	≤0.25	1	8	4	≤32	>38/2	0.5	*bla* $_{TEM}$
1EC87	2017	ETEC	>64	2	≤0.5	≤0.5	≤0.5	≤1	4/8.	≤0.5	≤0.25	≤0.25	0.5	2	0.5	≤32	>38/2	≤0.25	*bla* $_{TEM}$
1EC94	2017	ETEC	4	2	≤0.5	≤0.5	≤0.5	≤1	≤2/4	≤0.5	≤0.25	≤0.25	0.5	2	1	≤32	≤9.5/0.5	0.5	None
1EC99	2017	EHEC/ETEC	1	2	≤0.5	≤0.5	≤0.5	≤1	≤2/4	≤0.5	≤0.25	≤0.25	1	4	1	≤32	≤9.5/0.5	≤0.25	None
1EC110	2017	ETEC	>64	>16	>32	>16	>16	>4	8/16.	>16	≤0.25	≤0.25	1	8	>8	≤32	>38/2	>4	*bla* $_{TEM}$, *bla* $_{OXA-1}$-like , *bla* $_{CTX-M}$ group 1
1EC112	2017	ETEC	>64	8	≤0.5	≤0.5	≤0.5	≤1	>8/16	≤0.5	≤0.25	≤0.25	0.5	2	4	≤32	>38/2	≤0.25	*bla* $_{TEM}$
1EC119	2017	ETEC	1	1	≤0.5	≤0.5	≤0.5	≤1	≤2/4	≤0.5	≤0.25	≤0.25	0.5	2	1	≤32	≤9.5/0.5	≤0.25	None
1EC126	2017	ETEC	>64	2	≤0.5	≤0.5	≤0.5	≤1	4/8 .	≤0.5	≤0.25	≤0.25	1	4	8	≤32	>38/2	≤0.25	*bla* $_{TEM}$
1EC134	2017	ETEC	>64	>16	>32	16	>16	>4	8/16.	>16	≤0.25	≤0.25	2	8	8	≤32	>38/2	0.5	*bla* $_{TEM}$, *bla* $_{CTX-M}$ group 1
1EC143	2017	ETEC	1	2	≤0.5	≤0.5	≤0.5	≤1	≤2/4	≤0.5	≤0.25	≤0.25	1	8	1	≤32	≤9.5/0.5	0.5	None
1EC165	2017	ETEC	>64	2	≤0.5	≤0.5	≤0.5	≤1	8/16.	≤0.5	≤0.25	≤0.25	1	4	>8	≤32	>38/2	≤0.25	*bla* $_{TEM}$
1EC167	2017	ETEC	1	1	≤0.5	≤0.5	≤0.5	≤1	≤2/4	≤0.5	≤0.25	≤0.25	2	8	4	≤32	>38/2	0.5	None
1EC176	2017	EPEC	>64	>16	>32	16	16	>4	8/16.	>16	≤0.25	≤0.25	1	16	>8	≤32	>38/2	>4	*bla* $_{OXA-1}$-like , *bla* $_{CTX-M}$ group 1
1EC178	2017	ETEC	>64	>16	32	8	4	>4	>8/16	16	≤0.25	≤0.25	1	8	>8	≤32	>38/2	>4	*bla* $_{TEM}$
1EC179	2018	ETEC	1	1	≤0.5	≤0.5	≤0.5	≤1	≤2/4	≤0.5	≤0.25	≤0.25	0.5	≤1	4	≤32	>38/2	≤0.25	None
1EC183	2018	EPEC	4	2	≤0.5	≤0.5	≤0.5	≤1	≤2/4	≤0.5	≤0.25	≤0.25	1	4	2	≤32	>38/2	≤0.25	None
1EC187	2018	ETEC	>64	>16	>32	16	>16	>4	>8/16	>16	≤0.25	≤0.25	4	4	>8	≤32	>38/2	>4	*bla* $_{TEM-1B}$, *bla* $_{OXA-1}$, *bla* $_{OXA-181}$ *bla* $_{CTX-M-15}$
1EC188A	2018	ETEC	>64	>16	≤0.5	≤0.5	≤0.5	≤1	8/16 .	≤0.5	≤0.25	≤0.25	0.5	2	2	≤32	>38/2	≤0.25	*bla* $_{TEM}$
1EC188B	2018	EPEC	>64	2	≤0.5	≤0.5	≤0.5	≤1	≤2/4	≤0.5	≤0.25	≤0.25	0.5	≤1	2	≤32	>38/2	≤0.25	*bla* $_{TEM}$
1EC190A	2018	ETEC	1	2	≤0.5	≤0.5	≤0.5	≤1	≤2/4	≤0.5	≤0.25	≤0.25	0.5	2	0.5	≤32	>38/2	<0.5	None
1 EC190B	2018	EIEC	1	1	≤0.5	≤0.5	≤0.5	≤1	≤2/4	≤0.5	≤0.25	≤0.25	0.5	4	4	≤32	>38/2	≤0.25	None
1EC194A	2018	ETEC	>64	1	≤0.5	≤0.5	≤0.5	≤1	≤2/4	≤0.5	≤0.25	≤0.25	2	2	2	≤32	>38/2	≤0.25	*bla* $_{TEM}$
1EC194B	2018	EAEC	>64	2	≤0.5	≤0.5	≤0.5	≤1	4/8.	≤0.5	≤0.25	≤0.25	0.5	2	2	≤32	>38/2	≤0.25	*bla* $_{TEM}$
1 EC195	2018	ETEC	>64	2	2	≤0.5	≤0.5	≤1	4/8.	≤0.5	≤0.25	≤0.25	0.5	2	4	≤32	>38/2	1	*bla* $_{TEM}$
1EC199	2018	EAEC	>64	>16	>32	16	>16	>4	8/16.	>16	≤0.25	≤0.25	0.5	4	>8	≤32	>38/2	1	*bla* $_{OXA-1}$-like , *bla* $_{CTX-M}$ group 1
1 EC200	2018	EAEC	>64	4	≤0.5	≤0.5	≤0.5	≤1	8/16.	≤0.5	≤0.25	≤0.25	0.5	2	4	≤32	>38/2	≤0.25	*bla* $_{TEM}$
1 EC201	2018	ETEC	>64	4	≤0.5	≤0.5	≤0.5	≤1	8/16.	≤0.5	≤0.25	≤0.25	0.5	4	4	≤32	>38/2	≤0.25	*bla* $_{TEM}$
1EC202	2018	ETEC	>64	>16	≤0.5	≤0.5	≤0.5	≤1	8/16.	≤0.5	≤0.25	≤0.25	0.5	2	>8	≤32	>38/2	≤0.25	*bla* $_{TEM}$
1EC207	2018	EAEC	>64	2	≤0.5	≤0.5	≤0.5	≤1	4/8.	≤0.5	≤0.25	≤0.25	2	8	8	≤32	>38/2	0.5	*bla* $_{TEM}$
1EC214	2018	EAEC	>64	>16	>32	8	>16	>4	8/16.	16	≤0.25	≤0.25	0.5	2	4	≤32	>38/2	≤0.25	*bla* $_{TEM}$, *bla* $_{CTX-M}$ group 1
2EC9A	2017	ETEC	>64	4	≤0.5	≤0.5	≤0.5	≤1	8/16.	≤0.5	≤0.25	≤0.25	1	2	>8	≤32	>38/2	≤0.25	*bla* $_{TEM}$
2EC9B	2017	ETEC	>64	4	≤0.5	≤0.5	≤0.5	≤1	8/16.	≤0.5	≤0.25	≤0.25	1	4	2	≤32	>38/2	≤0.25	*bla* $_{TEM}$
1EC38	2016	Non-DEC	4	> 16	≤0.5	≤0.5	≤0.5	≤1	8 /16.	≤0.5	≤0.25	≤0.25	0.5	4	2	≤32	≤9.5/0.5	≤0.25	None
1EC55	2016	Non-DEC	> 64	>16	≤0.5	≤0.5	≤0.5	≤1	8/16.	≤0.5	≤0.25	≤0.25	0.5	2	8	≤32	>38/2	≤0.25	*bla* $_{TEM}$
1EC71	2016	Non-DEC	1	2	≤0.5	≤0.5	≤0.5	≤1	≤2/4	≤0.5	≤0.25	≤0.25	1	2	2	≤32	≤9.5/0.5	≤0.25	None
1EC81	2017	Non-DEC	>64	2	≤0.5	≤0.5	≤0.5	≤1	8/16.	≤0.5	≤0.25	≤0.25	1	4	> 8	≤32	>38/2	≤0.25	*bla* $_{TEM}$
1EC82	2017	Non-DEC	4	2	≤0.5	≤0.5	≤0.5	≤1	≤2/4	≤0.5	≤0.25	≤0.25	0.5	4	4	≤32	>38/2	≤0.25	None
1EC90	2017	Non-DEC	>64	16	≤0.5	≤0.5	≤0.5	≤1	8/16.	≤0.5	≤0.25	≤0.25	≤0.25	≤1	>8	≤32	>38/2	≤0.25	*bla* $_{TEM}$
1EC91	2017	Non-DEC	>64	16	≤0.5	≤0.5	≤0.5	≤1	>8/16	≤0.5	≤0.25	≤0.25	0.5	4	2	≤32	>38/2	1	*bla* $_{TEM}$
1EC103	2017	Non-DEC	>64	2	≤0.5	≤0.5	≤0.5	≤1	8 /16.	≤0.5	≤0.25	≤0.25	0.5	4	4	≤32	≤9.5/0.5	≤0.25	*bla* $_{TEM}$
1EC111	2017	Non-DEC	> 64	>16	>32	16	8	>4	4/ 8.	>16	≤0.25	≤0.25	1	8	>8	≤32	>38/2	0.5	*bla* $_{OXA-1}$-like , *bla* $_{CTX-M}$ group 1
1EC122	2017	Non-DEC	> 64	4	≤0.5	≤0.5	≤0.5	≤1	4/ 8.	≤0.5	≤0.25	≤0.25	0.5	2	>8	≤32	>38/2	1	*bla* $_{TEM}$
1EC131	2017	Non-DEC	>64	4	≤0.5	≤0.5	≤0.5	≤1	8/ 16.	≤0.5	≤0.25	≤0.25	1	4	8	≤32	>38/2	1	*bla* $_{TEM}$
1EC139	2017	Non-DEC	> 64	2	≤0.5	≤0.5	≤0.5	≤1	8/ 16.	≤0.5	≤0.25	≤0.25	0.5	≤1	2	≤32	>38/2	≤0.25	None
1EC144	2017	Non-DEC	>64	2	≤0.5	≤0.5	≤0.5	≤1	4/ 8.	≤0.5	≤0.25	≤0.25	0.5	≤1	2	≤32	>38/2	≤0.25	*bla* $_{TEM}$
1EC168	2017	Non-DEC	1	2	≤0.5	≤0.5	≤0.5	≤1	≤2/4	≤0.5	≤0.25	≤0.25	0.5	2	4	≤32	>38/2	≤0.25	None
1EC169	2017	Non-DEC	1	2	≤0.5	≤0.5	≤0.5	≤1	≤2/4	≤0.5	≤0.25	≤0.25	0.5	2	4	≤32	≤9.5/0.5	≤0.25	None
1EC177	2017	Non-DEC	4	2	≤0.5	≤0.5	≤0.5	≤1	≤2/4	≤0.5	≤0.25	≤0.25	1	≤1	>8	≤32	≤9.5/0.5	≤0.25	None
1EC181	2018	Non-DEC	>64	4	≤0.5	≤0.5	≤0.5	≤1	8/16.	≤0.5	≤0.25	≤0.25	0.5	2	>8	≤32	38/2.	1	*bla* $_{TEM}$
1EC192	2018	Non-DEC	> 64	2	≤0.5	≤0.5	≤0.5	≤1	4/8.	≤0.5	≤0.25	≤0.25	≤0.25	≤1	4	≤32	>38/2	0.5	*bla* $_{TEM}$
1EC196	2018	Non-DEC	>64	>16	32	8	8	>4	4/8.	16	≤0.25	≤0.25	0.5	4	2	≤32	≤9.5/0.5	1	*bla* $_{CTX-M}$ group 1
1EC197	2018	Non-DEC	>64	16	≤0.5	≤0.5	≤0.5	≤1	>8/16	≤0.5	≤0.25	≤0.25	0.5	2	2	≤32	>38/2	1	*bla* $_{TEM}$
1EC198A	2018	Non-DEC	>64	4	≤0.5	≤0.5	≤0.5	≤1	4/8.	≤0.5	≤0.25	≤0.25	0.5	4	8	≤32	>38/2	≤0.25	*bla* $_{TEM}$
1EC198B	2018	Non-DEC	>64	2	≤0.5	≤0.5	≤0.5	≤1	8/16 .	≤0.5	≤0.25	≤0.25	1	2	4	≤32	>38/2	≤0.25	*bla* $_{TEM}$
1EC213	2018	Non-DEC	>64	8	≤0.5	≤0.5	≤0.5	≤1	>8/16	≤0.5	0.5	≤0.25	0.5	16	8	≤32	>38/2	4	*bla* $_{TEM-35}$, *bla* $_{OXA-1}$ *bla* $_{OXA-181}$
1EC215	2018	Non-DEC	>64	>16	>32	>16	>16	>4	8/16.	>16	≤0.25	≤0.25	>8	32	>8	≤32	>38/2	>4	*bla* $_{TEM}$, *bla* $_{OXA-1}$-like
1EC217	2018	Non-DEC	1	1	≤0.5	≤0.5	≤0.5	≤1	≤2/4	≤0.5	≤0.25	≤0.25	1	2	4	≤32	>38/2	≤0.25	None

Supplementary dataset 2

STRAIN ID	ACCESSION NUMBER	COUNTRY	NDM	OTHER CARBAPENEMASES	CTX-M
1EC187 (This study)	CP061108	GHANA		OXA-181	CTX-M-15
Reference	CP034958	China	NDM-5		CTX-M-15
	ERR1197967	Germany	-	-	CTX-M-15
	ERR1195674	Germany	-	-	CTX-M-15
	ERR1197947	Germany	-	-	CTX-M-15
	ERR1197948	Germany	-	-	CTX-M-15
	ERR2019177	Hungary	-	-	CTX-M-15
	ERR2019135	Czech Republic	-	-	CTX-M-15
	ERR1197950	Germany	-	-	CTX-M-15
	ERR1197949	Germany	-	-	CTX-M-15
	ERR1619455	Netherlands	-	-	CTX-M-15
	ERR2223765	Poland	-	-	CTX-M-15
	ERR712775	Germany	-	-	CTX-M-15
	ERR2116736	Germany	-	-	CTX-M-15
	ERR1415483	Romania	-	-	CTX-M-15
	ERR1619351	Netherlands	-	-	CTX-M-15
	ERR1619365	Netherlands	-	-	CTX-M-15
	SRR4017857	United States	-	-	-
	SRR2724080	Norway	NDM-1	-	-
	SRR3290089	United States	-	-	CTX-M-15
	ERR1891337	Africa	-	-	CTX-M-15
	ERR2019239	Slovenia	-	-	CTX-M-15
	ERR2019230	Romania	-	-	CTX-M-32
	ERR2138622	-	-	-	-
	4SRR7026307	China	NDM-1	-	CTX-M-15
	SRR7026287	China	NDM-5	-	-
	ERR1946900	Philippines	NDM-4	-	CTX-M-15
	SRR7026311	China	NDM-5	-	-
	SRR3051062	United Kingdom	-	OXA-181	CTX-M-15
	ERR1946929	Philippines	NDM-1	OXA-181	CTX-M-15
	ERR1946930	Philippines	NDM-1	OXA-181	CTX-M-15
	ERR1946920	Philippines	NDM-1	OXA-181	CTX-M-15
	SRR5714046	Canada	-	OXA-181	CTX-M-15
	SRR5312143	United States	-	OXA-181	CTX-M-15
	ERR1217055	Norway	-	OXA-181	CTX-M-15
	SRR5714073	Canada	-	OXA-181	-
	ERR1415560	United Kingdom	-	OXA-181	-
	SRR3051068	United Kingdom	-	OXA-181	CTX-M-15
	ERR1541417	Turkey	-	OXA-181	CTX-M-15
	ERR1218627	Thailand	-	-	CTX-M-15
	SRR6442662	China	NDM-5	-	CTX-M-65
	SRR5942764	Thailand	-	OXA-232	CTX-M-14, CTX-M-15
	SRR6474925	China	-	OXA-181	CTX-M-15
	SRR7026304	China	-	OXA-181	CTX-M-15
	ERR1971612	Denmark	-	OXA-181	CTX-M-15
	SRR7716572	China	NDM-5	-	-
	ERR766384	-	-	OXA-181	CTX-M-15
	ERR1374952	Europe	-	OXA-181	CTX-M-15
	ERR1971583	Denmark	-	OXA-181	-
	ERR2088799	Italy	-	OXA-181	CTX-M-15
	ERR1946916	Philippines	NDM-1	-	CTX-M-15
	ERR1946915	Philippines	-	-	-
	ERR1946913	Philippines	NDM-1	-	CTX-M-15
	ERR1946910	Philippines	NDM-1	-	CTX-M-15
	ERR1946914	Philippines	NDM-1	-	CTX-M-15
	ERR1971544	Denmark	-	OXA-181	-
	ERR1359224	Germany	-	-	CTX-M-15
	ERR1218603	Thailand			CTX-M-55
	ERR1218604	Thailand			CTX-M-55
	ERR1218610	Thailand			CTX-M-55
	ERR1218630	Thailand			CTX-M-15
	ERR1218707	Thailand			CTX-M-55
	ERR1218718	Thailand			CTX-M-15
	ERR2060058	Vietnam			CTX-M-55
	ERR2060120	Vietnam			CTX-M-55
	SRR3999080	USA	-	KPC-3	-
	SRR3999074	USA	-	KPC-3	-
	SRR3987591	Saudi Arabia	-	-	-
	SRR3987983	USA	-	-	-
	SRR4262794	USA	-	-	-
	SRR4262712	USA	-	-	-
	SRR1178257	USA	-	-	-
	SRR5470036	USA	-	-	-
	SRR6942787	China	NDM-5	-	CTX-M-15
	SRR5714064	Canada		OXA-181	CTX-M-15
