# Supplementary figures and images for: Emergence of oxacillinase-181 carbapenemase-producing diarrheagenic Escherichia coli in Ghana
Source: Emerg Microbes Infect. 2021 May 4;10(1):865–73. doi: 10.1080/22221751.2021.1920342 (PMC8110189; doi:10.1080/22221751.2021.1920342)

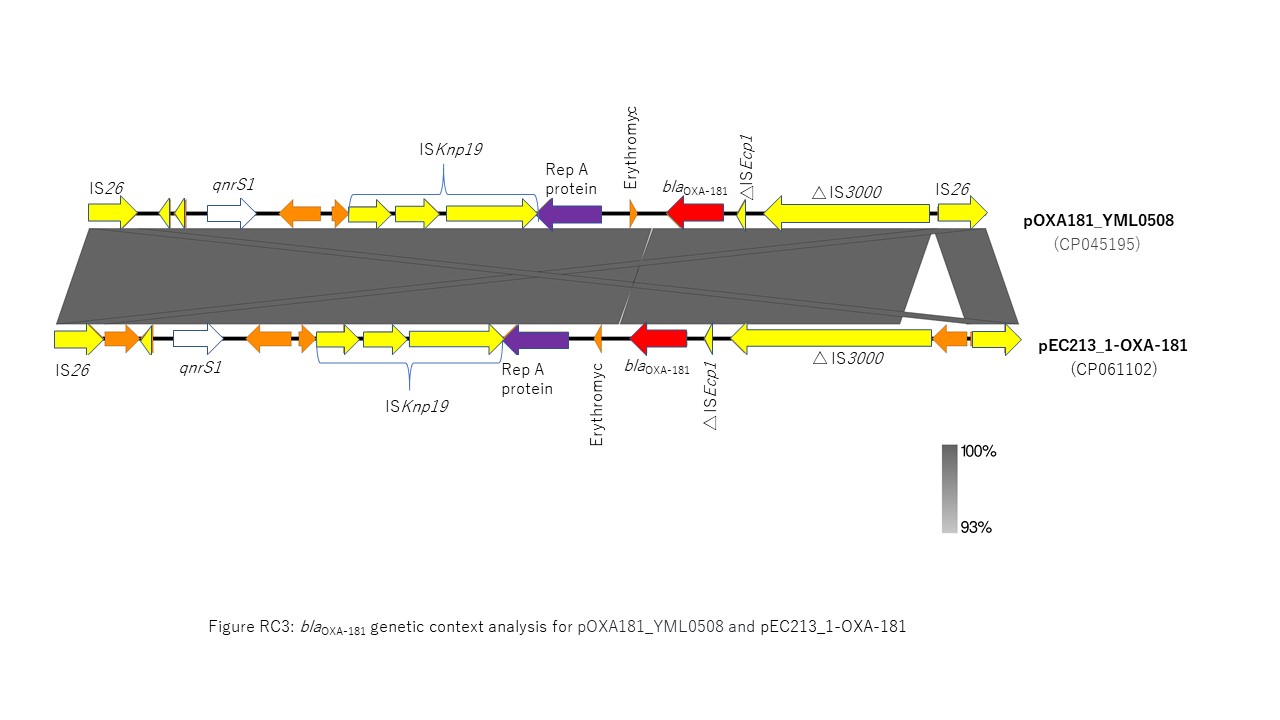

Supplement: Figure_RC3.jpg [file TEMI_A_1920342_SM6444.jpg]

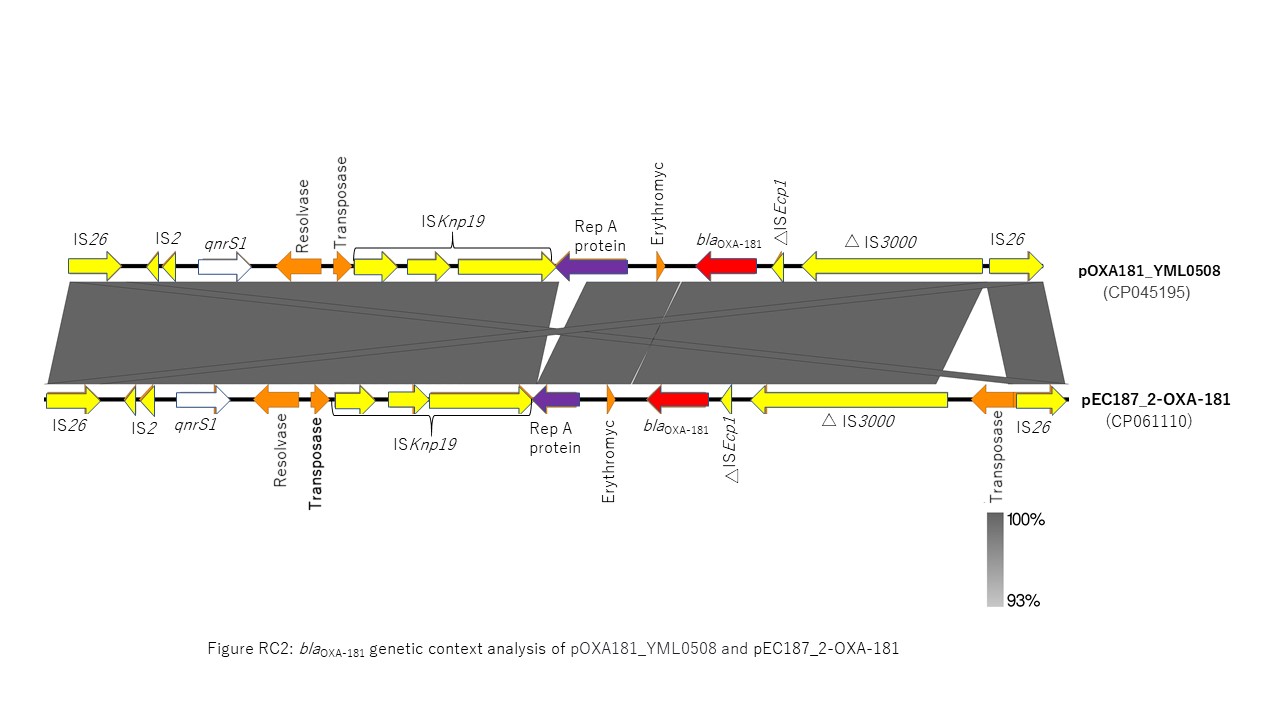

Supplement: Figure_RC2.jpg [file TEMI_A_1920342_SM6443.jpg]

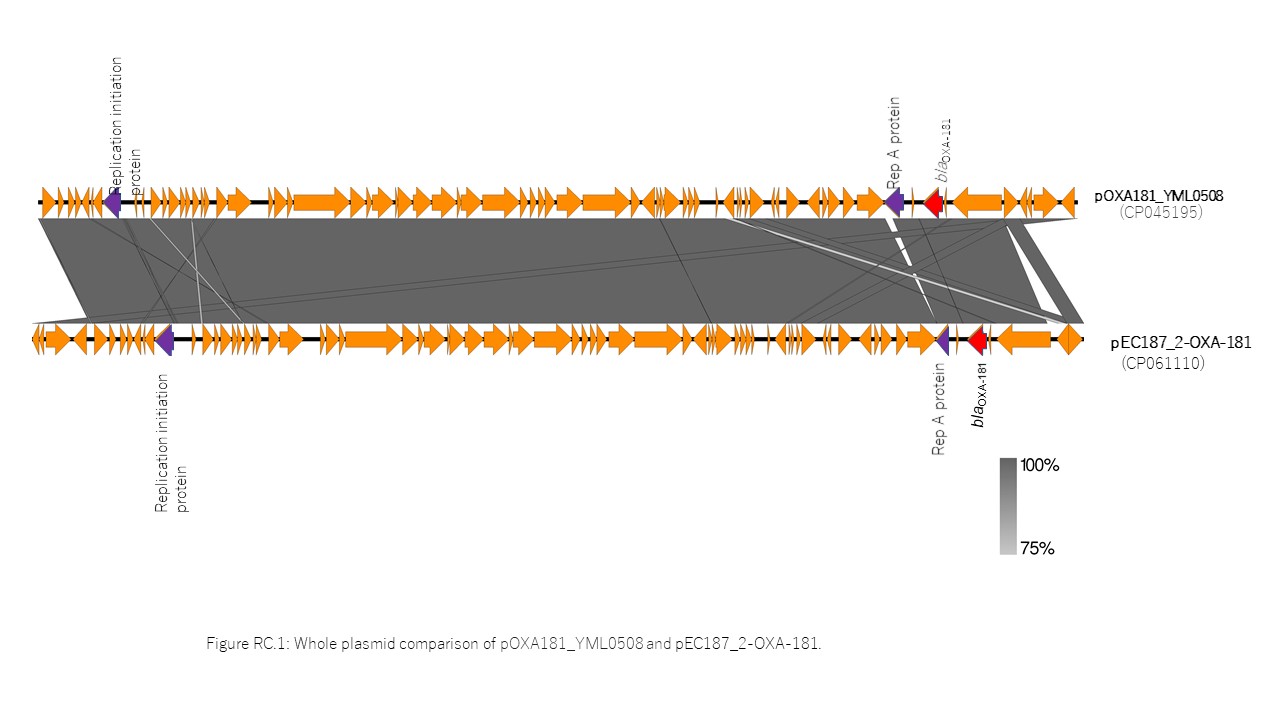

Supplement: Figure_RC.1.jpg [file TEMI_A_1920342_SM6442.jpg]
